# Integrative genomic and molecular dynamics characterisation of *acrB*_R717L/Q mutations in azithromycin-resistant *Salmonella* Typhi from India

**DOI:** 10.3389/fmicb.2026.1837537

**Published:** 2026-06-29

**Authors:** Tharani Priya Thirumoorthy, Jobin John Jacob, Nirmaladevi Ponnusamy, R. Subbulakshmi, Pavithra Sathya Narayanan, Monisha Priya Teekaraman, U. Yamini, Gunachandru Raja, Rosemol Nelson, Jacob John, Kamini Walia, Balaji Veeraraghavan

**Affiliations:** 1 Department of Clinical Microbiology, Christian Medical College, Vellore, Tamil Nadu, India; 2 The Tamil Nadu Dr.M.G.R Medical University, Chennai, Tamil Nadu, India; 3 Department of Community Health, Christian Medical College, Vellore, Tamil Nadu, India; 4 Descriptive Research Division, Indian Council of Medical Research, New Delhi, India

**Keywords:** *Salmonella* Typhi, typhoid fever, azithromycin-resistance, whole genome sequencing, molecular dynamics simulation

## Abstract

**Background:**

Azithromycin remains a highly effective oral therapy for uncomplicated typhoid fever caused by extensively drug-resistant (XDR) *Salmonella* Typhi strains. However, azithromycin resistance has been reported in low and middle-income countries (LMICs). In this study, we identified three azithromycin-resistant *S.* Typhi isolates and performed phylogenetic analysis along with molecular dynamics simulation to assess the structural stability and conformational dynamics of the *acrB* efflux pump harbouring R717L/Q mutations.

**Methods:**

Antimicrobial susceptibility of three azithromycin-resistant *S.* Typhi isolates was determined using the minimum inhibitory concentration (MIC) assay following CLSI guidelines. Whole genome sequencing was performed to elucidate the molecular determinants of resistance and to assess phylogenetic relatedness within a global genomic framework. In addition, computational analysis was conducted to evaluate the impact of mutations on drug resistance.

**Results:**

All three azithromycin-resistant *S.* Typhi exhibited an MIC value of 32 mg/L. Phylogenetic analysis demonstrated that two isolates clustered within a distinct sublineage (genotype 4.3.1.2.1) of lineage II (4.3.1.2), whereas the third isolate belonged to lineage 4.3.1.3, consistent with the patient’s geographic link to Bangladesh. Antimicrobial resistance gene analysis identified the *acrB*_R717Q mutation in the two isolates and *acrB*_R717L in the single isolate, both associated with azithromycin resistance. Molecular dynamics simulations indicated greater structural deviation in the R717L variant compared to R717Q, with possible increased conformational flexibility that could influence efflux pump dynamics.

**Conclusion:**

Our findings underscore the need for genomic surveillance frameworks to incorporate both plasmid-mediated resistance genes (e.g., *mphA*) and chromosomal mutations, such as *acrB*_R717L/Q variants, to effectively track emerging azithromycin resistance and guide typhoid conjugate vaccine deployment alongside antibiotic stewardship.

## Introduction

Typhoid and paratyphoid fever, collectively known as enteric fever, are caused by infection with the human-restricted pathogen *Salmonella* Typhi and *Salmonella Paratyphi* serovars A, B, and C (Hooda et al., 2019). The disease is mainly transmitted through contaminated food and water infected by human faeces (Basnyat et al., 2021). According to the 2021 Global Burden of Diseases study, enteric fever accounted for an estimated 9.3 million cases and 107.5 thousand deaths worldwide, with the highest burden observed in South Asia (Piovani et al., 2024). However, endemicity persists in low- and middle-income countries due to escalating antimicrobial resistance and inadequate infrastructure, including poor sanitation and limited access to safe water.

Antimicrobial therapy is the mainstay of treatment for typhoid fever. Nevertheless, the overuse of antibiotics drives antimicrobial resistance in typhoidal *Salmonella* through horizontal gene transfer and point mutations (Kirchhelle et al., 2019; Chavan and Angadi, 2025). Over the past decades, the emergence of multidrug-resistant *Salmonella* Typhi carrying an IncHI plasmid necessitated the shift toward fluoroquinolones as first-line therapy. However, the subsequent rise of fluoroquinolone-non-susceptible *S.* Typhi in the early 2000’s led to the use of third-generation cephalosporins as the drug of choice. In 2016, the emergence of an extensively drug-resistant *S.* Typhi outbreak in Pakistan, associated with an IncY plasmid, further restricted the treatment options, leaving azithromycin as the only effective oral drug. Consequently, the current guidelines recommend azithromycin as empirical therapy for uncomplicated XDR typhoid fever and carbapenems for complicated infections (Nabarro et al., 2022). Alarmingly, azithromycin resistance in *S.* Typhi has been reported in Bangladesh, Nepal, Pakistan, and India. The primary resistance mechanism underlying azithromycin in clinical isolates of *S.* Typhi is linked to a missense or non-synonymous mutation in the efflux pump *acrB*-R717L/Q (Carey et al., 2021; Duy et al., 2020). Notably, sporadic cases of acquired azithromycin-resistant *S.* Typhi carrying *mphA* have been documented in Bangladesh and the United States (Dola et al., 2022; Tagg et al., 2024). The escalating antimicrobial resistance in *S.* Typhi, including resistance to last-resort antibiotics, threatens treatment strategies and necessitates sustained genomic surveillance to monitor resistance epidemiology and inform effective local and national treatment guidelines.

Mutations in the *acrB* gene (particularly R717) have been associated with azithromycin resistance, yet their structural impact remains unknown. These substitutions, such as R717L/Q likely alter interactions, potentially affecting stability and conformational dynamics essential for efflux. Molecular dynamics simulations will elucidate changes in protein flexibility and reveal how R717 variants influence azithromycin translocation efficiency.

This study provides additional insights by integrating genomic analysis with molecular dynamics simulations to investigate azithromycin resistance in *S.* Typhi. Using whole-genome sequencing (WGS)-based genomic surveillance, we monitor high-risk circulating genotypes and antimicrobial resistance trends in *S.* Typhi. We further report azithromycin-resistant *S.* Typhi and employ comparative genomic analysis to investigate the emergence and spread of resistance, complemented by molecular dynamics simulation to assess the impact of *acrB* mutations. Overall, our findings underscore the importance of genomic surveillance for early outbreak detection and evidence-based public health interventions.

## Materials and methods

### Study design

The study included culture-confirmed, non-repetitive *S.* Typhi isolates obtained from blood cultures of patients with febrile illness at the Department of Clinical Microbiology, Christian Medical College and Hospital, Vellore, collected during routine enteric fever screening from January 2023 to December 2024.

### Ethical approval

The study was approved by the Institutional Review Board (IRB) of Christian Medical College, Vellore (IRB Min No. 15247 dated 22-03-2023).

### Bacterial identification and antimicrobial susceptibility testing

Bacterial isolates were confirmed as *S.* Typhi using standard biochemical tests and slide agglutination with commercial antisera (BD Difco, USA) as per the manufacturer’s instructions. Antimicrobial susceptibility testing was performed using the Kirby-Bauer disk diffusion method against ampicillin, chloramphenicol, co-trimoxazole, ciprofloxacin, ceftriaxone and azithromycin. Minimum inhibitory concentrations (MIC) were determined using the broth microdilution (BMD) method against ciprofloxacin, ceftriaxone and azithromycin following CLSI guidelines (Clinical and Laboratory Standards Institute, 2024).

### DNA extraction and whole-genome sequencing

Genomic DNA was extracted using the QIAamp Mini Kit (QIAGEN, Germany) following the manufacturer’s instructions. Extracted DNA samples were quantified using Nanodrop One and Qubit Fluorometer and stored at −80 °C before use. Genomic DNA libraries were prepared using Illumina Nextera DNA Flex Library Kit and Nextera DNA CD Indexes (Illumina, Massachusetts, to yield 2MA, USA) for short-read sequencing on the Illumina Novaseq 6000 platform, yielding 2 × 150 bp paired-end reads. Raw reads underwent quality control, adapter trimming, and filtering to retain high-quality sequences (Phred score > 30), which were then assembled using SKESA V.3.0.0^1^ (Souvorov et al., 2018). *S.* Typhi serotyping was performed using SISTR (Yoshida et al., 2016), and genotypes were assigned using the GenoTyphi framework^2^ (Wong et al., 2016). Antimicrobial resistance genes were also detected using the AMRFinderPlus database v3.11.14 (Feldgarden et al., 2021).

### Phylogenetic analysis

To assess the phylogeny, the study isolates (*n* = 3) and representative global *S.* Typhi isolates (*n* = 160) (Supplementary Table S1) were mapped to *Salmonella* Typhi CT18 using snippy. Recombination regions were masked from the whole-genome single-nucleotide polymorphism (SNP) alignment with Gubbins (Croucher et al., 2015). A maximum phylogenetic tree was constructed using RAxML-NG v1.2.1 (Kozlov et al., 2019) under the GTR + GAMMA model with 1,000 bootstraps and visualised using Interactive Tree of Life software (iTOL v7.5) (Letunic and Bork, 2024).

### Molecular dynamics simulation

The *acrB* protein sequence of *Salmonella* Typhi (UniProtKB: Q8Z8T8) was retrieved, and no experimentally determined three-dimensional structure for the *acrB* protein was available in the Protein Data Bank (PDB). Therefore, the corresponding three-dimensional structure was obtained from the AlphaFold database. The wild-type (WT) model was used as a template to generate the R717L and R717Q mutant models through computational mutagenesis using PyMOL v3.1.1, in which the target residue was substituted, and the side chains were repacked. The stereochemical quality of all models was evaluated using Ramachandran plot analysis.

The effects of the non-synonymous substitutions on protein stability were predicted using DynaMut (incorporating mCSM, SDM, DUET, and ENCoM), which integrates graph-based signatures, statistical potentials, and normal mode analysis (Rodrigues et al., 2018). Subsequently, molecular dynamics (MD) simulations of the WT and mutant *acrB* models were performed following the methodology described by Murzyn et al. (2005). The *acrB* structure was embedded in a membrane composed of palmitoyloleoyl phosphatidylethanolamine (POPE) and palmitoyloleoyl phosphatidylglycerol (POPG) at a 3:1 ratio using the Membrane Builder module of the CHARMM-GUI interface v.3.8 (Lee et al., 2016). Molecular dynamics (MD) simulations were performed using GROMACS 2024.2 with the CHARMM36m force field for proteins and CHARMM36 lipid parameters. All systems were solved using TIP3P water molecules, with 0.15 M KCl added to neutralise the system and mimic physiological ionic strength (Jiang et al., 2022). Energy minimisation was performed using the steepest descent algorithm to remove steric clashes and optimise initial conformations. Following minimisation, the systems were equilibrated using a multi-stage protocol. Initially, NVT (constant volume, temperature) equilibration was performed for 50,000 steps to adjust atomic velocities and stabilise temperature at 310 K. Subsequently, systems were equilibrated using a gradual release of positional restraints on protein atoms in the NPT ensemble at 310 K and 1 bar using the Nosé-Hoover thermostat and the semi-isotropic Parrinello-Rahman barostat. Long-range electrostatic interactions were treated using the Particle Mesh Ewald (PME) method with a real-space cutoff of 1.2 nm. All bonds involving hydrogen atoms were constrained using the Linear Constraint Solver (LINCS) algorithm, allowing a 2 fs integration timestep.

Subsequently, unrestrained 100 ns production MD simulations were performed for the wild-type and mutant (R717L and R717Q) systems. MD simulation was performed without independent simulation runs, as the 100 ns simulation period was sufficient to achieve structural stability. To strengthen the analysis, statistical summaries (mean ± standard deviation) were calculated for the wild-type and mutant systems. However, the absence of independent simulation runs should be considered a limitation of the study. Structural stability and conformational dynamics were evaluated using root mean square deviation (RMSD), root mean square fluctuation (RMSF), radius of gyration (Rg), and principal component analysis (PCA).

### Statistical analysis

Trajectory-derived data were analysed using mean ± standard deviation values calculated over the simulation period.

## Results

### Phenotypic characterisation

A total of three azithromycin-resistant *S.* Typhi isolates were identified during the study period (2023–2024). Antimicrobial susceptibility results showed that the two isolates (BA9204, BA11780) were susceptible to ampicillin, chloramphenicol, cotrimoxazole and ceftriaxone, while resistant to ciprofloxacin and azithromycin. The remaining isolate (BV338) showed resistance to ampicillin, ciprofloxacin and azithromycin but remained susceptible to other tested antimicrobials. The minimum inhibitory concentration of the tested antibiotics is presented in Table 1.

**Table 1.** Minimum inhibitory concentration and molecular fingerprint of azithromycin-resistant *Salmonella* Typhi isolates

Isolate ID & Accession no	Location	Lineage (Genotype)	Year	Acquired resistance		Chromosomal resistance		Minimum Inhibitory Concentration (mg/L)		
				AMR determinants	Plasmids	QRDR Mutations	*acrB* Mutations	CIP^**a**^	CTR^**a**^	AZI^**a**^
BA9204 (ERR15664400)	Vellore	4.3.1.2.1	2023	-	-	*gyrA*_S83F/D87N; *parC*_S80I	*acrB*_R717Q	16R	0.06S	32R
BA11780 (ERR15664399)	Vellore	4.3.1.2.1	2023	-	-	*gyrA*_S83F/D87N; *parC*_S80I	*acrB*_R717Q	16R	0.12S	32R
BV338 (ERR15664398)	Bangladesh	4.3.1.3. Bdq	2023	blaTEM-1D; qnrS, sul2, tetA(A)	IncFIB(K)	*gyrA*_S83Y	*acrB*_R717L	8R	0.12S	32R

CIP, Ciprofloxacin; CTR, Ceftriaxone; AZI, Azithromycin.

a Interpretation based on CLSI 2024 guidelines (Breakpoints for *Salmonella* and *Shigella* spp).

### Genotyping and antimicrobial determinants

The genomes of azithromycin-resistant *S.* Typhi were characterised using the GenoTyphi scheme. All three isolates belonged to the H58 lineage (4.3.1), with two isolates assigned to sublineage 4.3.1.2.1 and one isolate to sublineage 4.3.1.3.Bdq; epidemiological analysis indicated that the affected patient was from Bangladesh.

### SNP-based phylogenetic analysis of *Salmonella* Typhi

**Figure 1:**
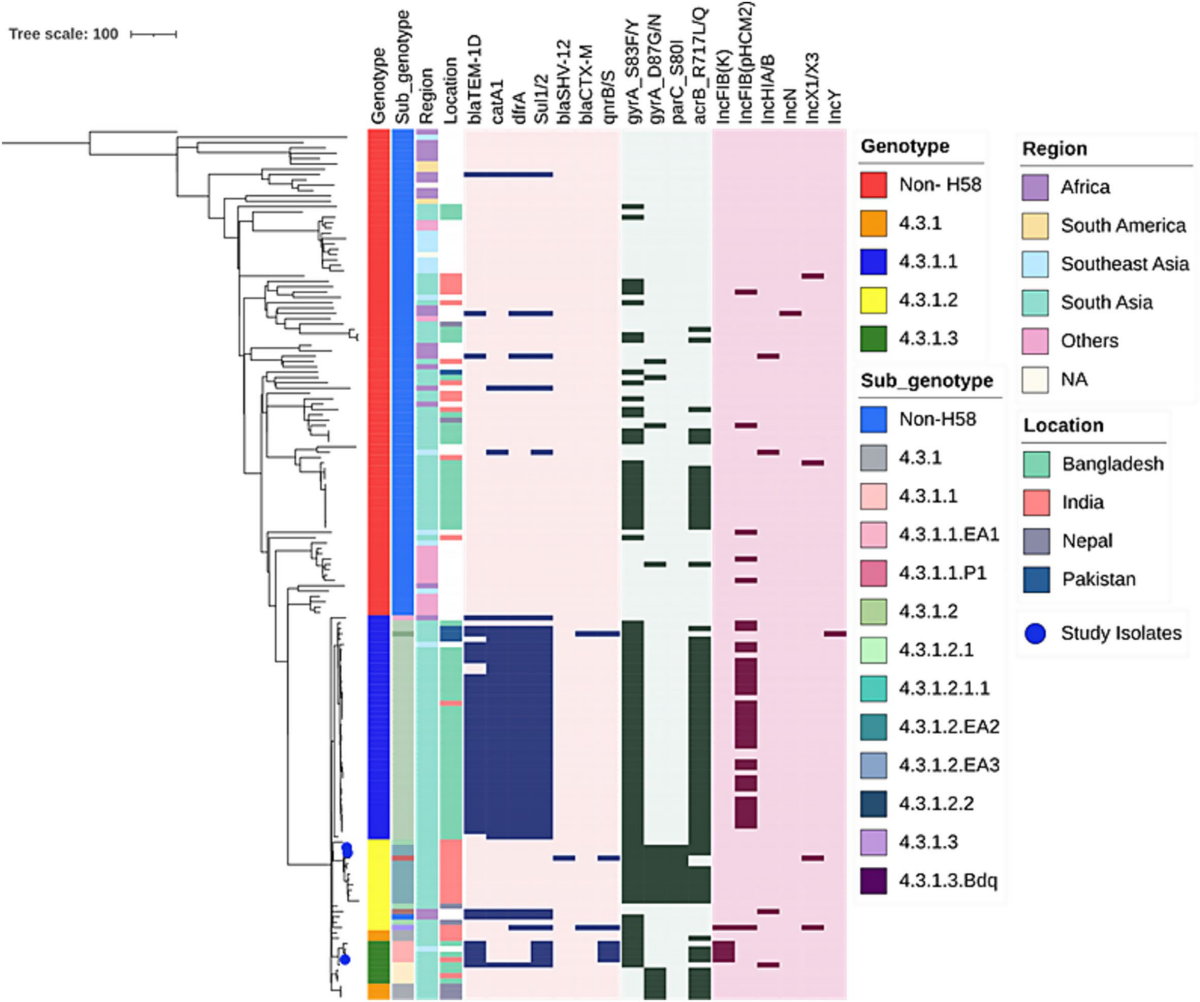
Maximum-likelihood phylogenetic tree based on core genome SNPs from 160 global H58 *Salmonella* Typhi isolates, mapped to reference *Salmonella* Typhi CT18 and rooted to an outgroup isolate (Genotype 4.3.1). Study isolates are marked with blue-coloured dots. The scale bar indicates the substitution per site. Coloured strips denote metadata: genotype (strip 1), subgenotype (strip 2), region (strip 3), and location (strip 4). The adjacent heatmap shows AMR genes, point mutations, and plasmids in H58 *Salmonella* Typhi isolates. Tree visualisation and annotation were performed using iTOL (https://itol.embl.de/).

A core genome SNP-based phylogenetic analysis was performed to determine the relatedness of the study isolates against a global collection of 160 genomes. The isolates showed no SNP differences relative to their closest lineages (4.3.1.2.1 and 4.3.1.2.1.1), whereas a divergence of two SNPs was observed relative to lineage II. The single isolate BV338 (ERR15664398) was assigned to lineage 4.3.1.3 and clustered within the 4.3.1.3.Bdq subclade (Figure 1). Antimicrobial resistance (AMR) gene profiling revealed that two isolates had triple QRDR mutations, which include *gyrA*_S83F/D87N, *parC*_S80I, hallmarks of the 4.3.1.2.1 lineage, along with the *acrB*_R717Q mutation. In contrast, the remaining isolate harboured an IncFIB(K) plasmid carrying *blaTEM-1D, qnrS, sul2, tetA(A),* along with a single QRDR mutation (*gyrA*_S83Y) and the *acrB*_R717L mutation. Notably, these *acrB*_R717L/Q mutations identified in H58 sublineages 4.3.1.2.1 (this study) suggest convergent evolution, similar to previously reported variants in India (4.3.1.2), Nepal (4.3.1), Bangladesh (4.3.1.1/4.3.1.3) and Pakistan (4.3.1.1) in the XDR clade. Phylogenetic analysis shows that these mutations are distributed across the distinct lineages rather than forming a single cluster, suggesting local independent acquisition under azithromycin pressure rather than clonal dissemination. This parallel emergence at codon 717 across both H58 and non-H58 lineages indicates *acrB* may represent a potential hotspot, warranting consideration of targeted surveillance beyond lineage-specific screening.

### Impact of mutations on protein dynamics

Ramachandran plot analysis of the wild-type (WT) and mutant structures (R717L and R717Q) established that 94.6% of residues were in favoured regions, 5.2% in allowed regions, 0.2% in generously allowed regions, and none in disallowed regions. To evaluate the impact of non-synonymous SNPs on *acrB* stability, we employed the DynaMut server. Both R717L and R717Q were predicted to be destabilising and to increase flexibility, suggesting possible functional alterations linked to antibiotic resistance (Table 2).

**Table 2:** Predicted effects of amino acid substitutions on the stability and flexibility of the *acrB* protein, calculated for each mutation relative to the wild type; ΔΔG values (kcal/mol) are reported, where negative values indicate destabilisation and positive values indicate stabilisation, along with the corresponding stability classification for each method.

ΔΔG (kcal/mol)						ΔΔS (kcal.mol^−1^. K^−1^)	
Mutation	mCSM	SDM	DUET	ENCoM	Predicted outcome	ENCoM	Predicted outcome
R717Q	−0.629	−0.910	−0.606	−0.327	Destabilising (−0.354)	0.408	Increase of molecule flexibility
R717L	−0.079	0.400	0.185	−0.247	Destabilising (−0.489)	0.309	Increase of molecule flexibility

**Figure 2:**
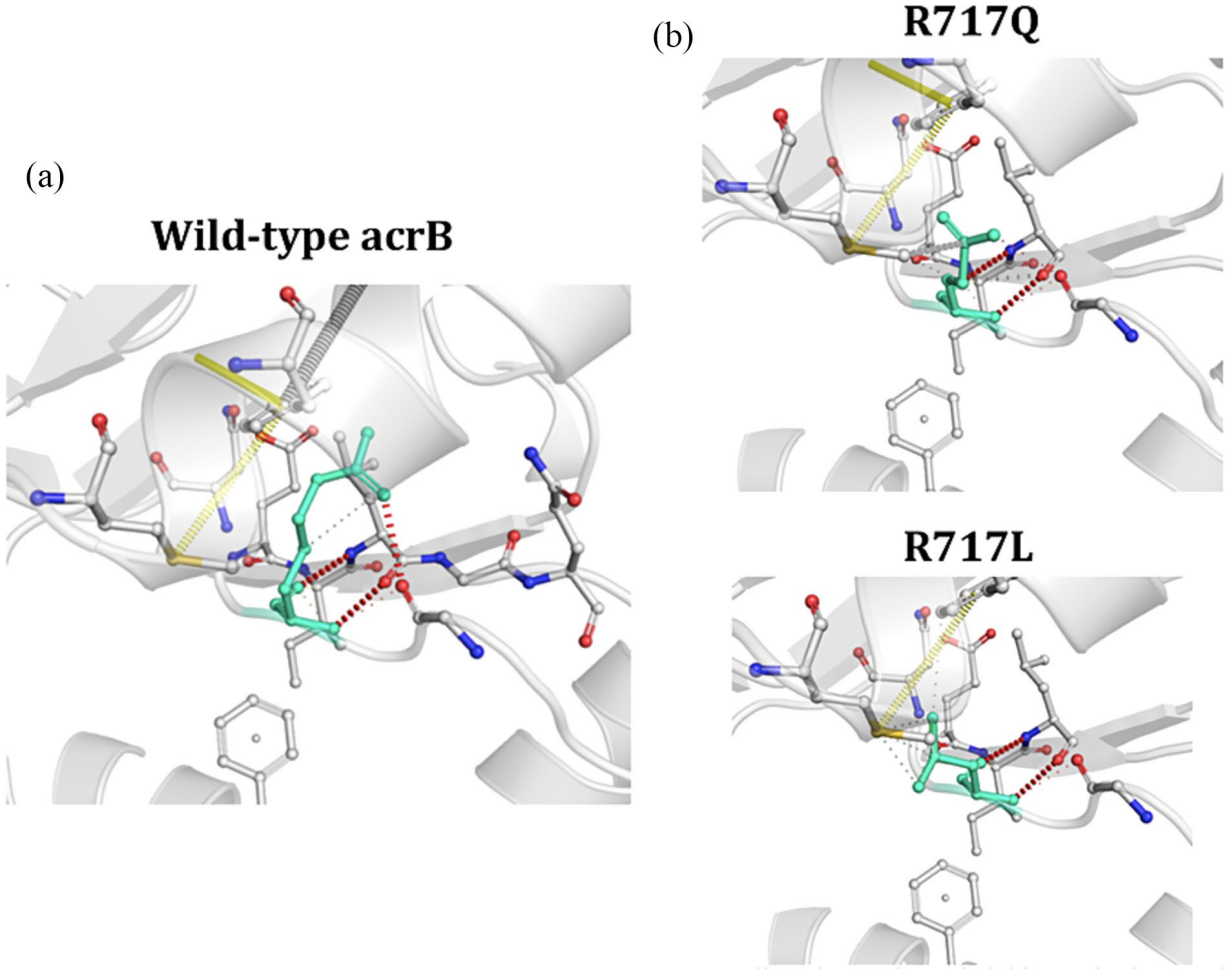
Effect of amino acid substitutions on interatomic interactions in the *acrB* protein: **(a)** Left, wild-type structure; **(b)** Right, top: R717Q (arginine to glutamine substitution at 717th position) and bottom: R717L (arginine to leucine substitution at 717th position); wild-type and mutant residues are shown in light grey and green, respectively, with interacting contacts indicated—polar interactions as red dashed lines, hydrophobic interactions in green, and weak hydrogen bonds in yellow.

The WT and mutant *acrB* structures were subjected to membrane-based MD simulations (Figure 2). Energy minimisation was performed using the steepest descent algorithm, yielding potential energies of −9.85×10^6^ kJ/mol (WT), −9.81×10^6^ kJ/mol (R717L), and −9.84×10^6^ kJ/mol (R717Q). To evaluate equilibration of the POPE–POPG bilayer environment, key system parameters were monitored from the start of the production simulations. Although the surface area per lipid is known to converge slowly during membrane MD simulations.

**Figure 3:**
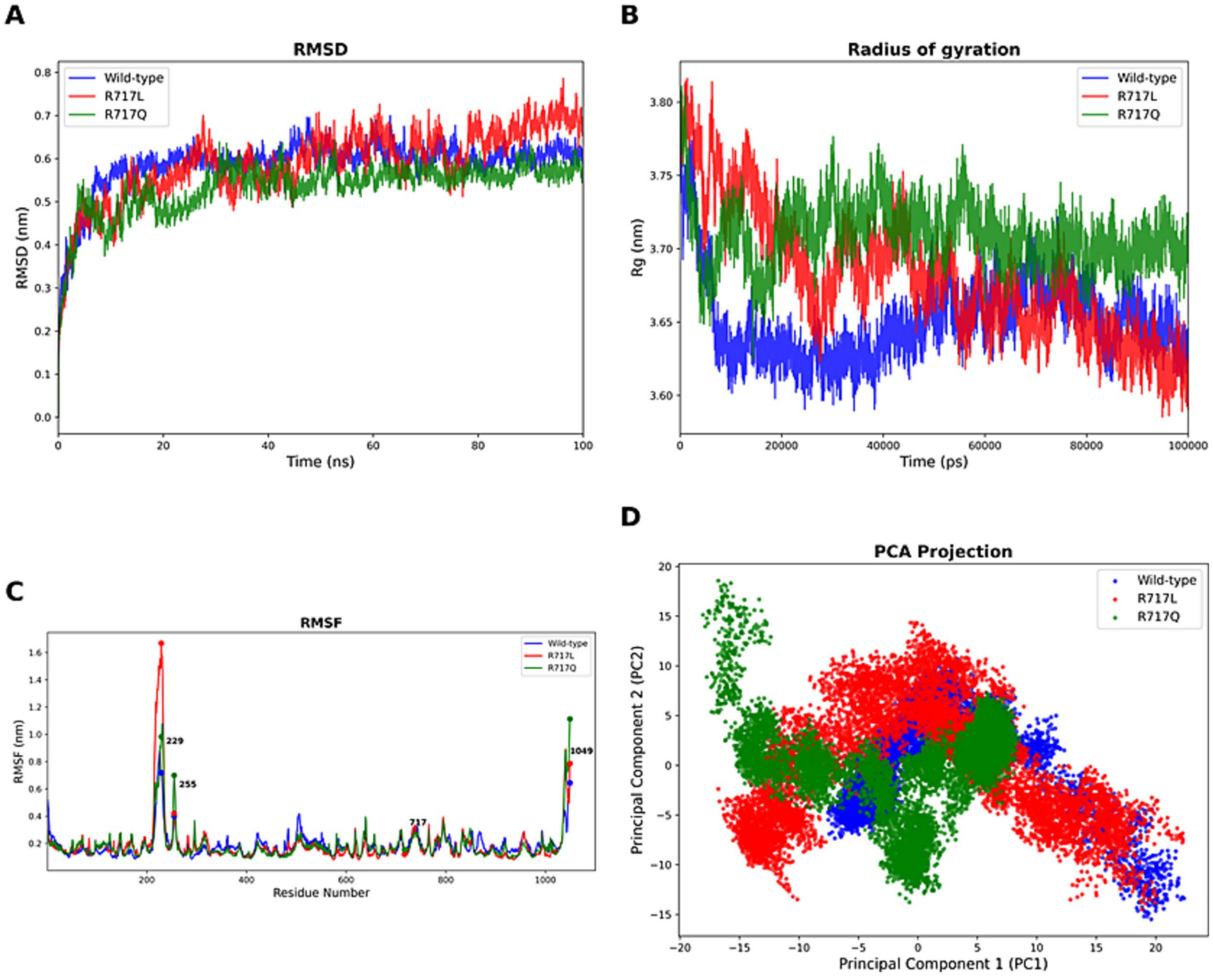
Molecular dynamics analysis of wild-type (WT) and mutant *acrB* proteins over a 100 ns simulation, comparing structural stability and conformational dynamics, including **(A)** Backbone RMSD profiles showing global stability, **(B)** Radius of gyration (Rg) indicating overall compactness, **(C)** RMSF highlighting residue-level flexibility with emphasis on loop regions, and **(D)** Principal component analysis (PCA) illustrating conformational sampling along PC1 and PC2.

Following confirmation of membrane equilibration, the structural stability of the WT and mutant models was evaluated using backbone RMSD analysis. RMSD values for all proteins increased to ~0.5 nm within the first 6 ns, consistent with the equilibration phase transitioning from the initial reference structure. All three systems showed a brief convergence at ~58 ns. The average RMSD values were WT (0.59 ± 0.06 nm), R717L (0.59 ± 0.08 nm), and R717Q (0.53 ± 0.06 nm). These observations suggest that R717Q may have minimal influence on *acrB* structural dynamics relative to WT, whereas R717L appears to exhibit greater conformational variability (Figure 3a).

### Protein compactness and residue-level flexibility assessment

The radius of gyration (Rg) was used to assess protein compactness. During the initial ~50 ns, all systems exhibited elevated Rg fluctuations, consistent with the equilibration phase. The average Rg values were 3.65 ± 0.03 nm (WT), 3.68 ± 0.04 nm (R717L) and 3.71 ± 0.02 nm (R717Q)(Figure 3b).

Residue-level RMSF analysis revealed minimal fluctuation at position 717. With WT and R717L both fluctuating at ~0.11 nm, while R717Q reached ~0.15 nm. The WT showed fluctuations of 0.50–0.85 nm at residues V225-G227 and S1045-R1049. In comparison, R717L exhibited higher fluctuations (1.00–1.66 nm at residues G217-S233 and 0.51–0.89 nm at residues S1037-R1049). Similarly, R717Q displayed enhanced fluctuations (0.51–1.07 nm at residues A216-I234 and 0.62–1.11 nm at residues E1038-R1049). The average RMSF values were comparable among WT (0.18 ± 0.09 nm), R717L (0.19 ± 0.18 nm), and R717Q (0.18 ± 0.12 nm). These observations suggest relatively increased flexibility in the loop region of R717L, whereas R717Q appeared to exhibit comparatively lower fluctuations (Figure 3c).

### Covariance matrix-based principal component analysis

Principal Component Analysis (PCA) was performed using the covariance matrix of Cα atoms to capture large-scale motions (Figure 3d). The diagonal elements of the matrix, which represent atomic fluctuations, were 129.987 nm^2^ for the WT, 207.607 nm^2^ for R717L, and 150.292 nm^2^ for R717Q, highlighting variant-specific differences in structural flexibility.

The conformational landscapes along PC1 and PC2 revealed that the WT occupied a compact space (PC1: −7.52 to 21.25 nm; PC2: −14.62 to 9.17 nm) consistent with stable motion. R717L sampled a broader range (PC1: −16.36 to 22.29 nm; PC2: −14.48 to 14.34 nm), indicative of greater flexibility and structural destabilisation. R717Q showed an intermediate distribution (PC1: −18.07 to 8.53 nm; PC2: −13.76 to 18.57 nm) reflecting conformational states distinct from both WT and R717L. The average PCA values were 1.01 × 10^−7^ ± 4.62 nm for WT, −2.61 × 10^−6^ ± 5.76 nm for R717L and 4.34 × 10^−7^ ± 0.02 nm for R717Q. Collectively, these results indicate that WT remains structurally stable, R717L drives destabilising motions, while R717Q exhibits an alternative conformational sampling.

## Discussion

The emergence of azithromycin resistance in *Salmonella* Typhi poses a significant threat to typhoid treatment, as it remains the only effective oral antibiotic with strong intracellular activity. Mutations such as *acrB*_R717L/Q and mobile resistance genes, including *mphA* and *ermB,* contribute to azithromycin resistance in *S.* Typhi, driven by selective pressure. Although the emergence of azithromycin resistance may be spontaneous, its early detection through WGS-based surveillance is essential for effective outbreak response and resistance containment. In this study, we integrate genomic analysis with MD simulation to explore the potential impact of these mutations.

During the study period, we identified three azithromycin-resistant *S.* Typhi isolates among high-risk circulating lineages. Azithromycin resistance was confirmed phenotypically by antimicrobial susceptibility testing and further validated at the genomic level using whole genome sequencing. We subsequently analysed the population structure of azithromycin-resistant *S.* Typhi isolates and examined the potential impact of *acrB*_R717L/Q substitutions on protein stability through computational approaches.

Notably, two isolates belonged to the 4.3.1.2.1 lineage and one to the 4.3.1.3.Bdq lineage, consistent with the patient’s epidemiological link to Bangladesh. The genotype 4.3.1.2.1 predominates in Indian surveillance studies. No SNP differences were observed between our azithromycin-resistant isolates and previously reported *S.* Typhi belonging to the same lineage, aside from the sole mutation in the *acrB* efflux pump. Collectively, these findings suggest that the azithromycin resistance emerges under selection pressure from increased azithromycin use during the COVID-19 pandemic, driving resistance in H58 lineage II. Moreover, the *S.* Typhi genome has become prone to acquiring plasmids from other Enterobacteriaceae, as reported by Tagg et al. (2024). The *mphA* gene was carried on plasmids across different lineages, indicating the independent emergence of resistant strains. Azithromycin-resistant strains have been reported in typhoid endemic countries, yet they occur in genetically distinct lineages. In Bangladesh, the *acrB* mutation was identified in 4.3.1.1 lineage, and in Pakistan, it occurred within the same lineage in the XDR clade. In contrast, isolates from India belonged to the 4.3.1.2 lineage, while those from Nepal clustered within 4.3.1.

Farishta et al. (2025) demonstrated that the *acrB*_R717L mutation plays a critical role in azithromycin resistance in Pakistan isolates. In this study, we extend this understanding by investigating additional mutations (leucine or glutamine) and their potential structural impact on *acrB*_R717L/Q. By integrating genomic analysis with molecular dynamics simulations, our computational predictions suggest that the *acrB*_R717L/Q mutations may be associated with altered structural stability and conformational dynamics of the *acrB* efflux pump, which could potentially influence efflux-related function. These observations suggest that single-residue substitutions at position 717 differentially modulate the global folding, flexibility and overall dynamics of *acrB*, potentially influencing its role in antibiotic resistance and contributing to reduced antibiotic susceptibility.

To translate these findings into clinical laboratory practice, we propose consideration of a structured diagnostic algorithm that may prioritise *acrB*_R717L/Q screening followed by plasmid-mediated genes such as *mphA* and *ermB*. An azithromycin MIC ≥32 mg/L plus H58 genotyping may warrant suspicion of *acrB*_R717 mutations, with WGS confirmation supporting a high likelihood of efflux-mediated azithromycin resistance. In resource-limited settings, targeted PCR or amplicon sequencing for the *acrB_R717 loci* and plasmid *mphA/ermB* genes may offer a pragmatic, cost-effective surrogate for WGS, enabling swift phenotypic-genotypic correlation and outbreak detection. Clinically, identification of *acrB_*R717L/Q in H58 *S.* Typhi may indicate high-level azithromycin resistance potential (MIC ≥ 32 mg/L), warranting referral to reference laboratories for confirmatory susceptibility testing. Furthermore, reference laboratories should consider flagging these variants in WGS reports to support clinical decision-making and promote rational evidence-based antibiotic use, thereby helping to preserve key therapeutic options for typhoid management.

A major limitation of this study is the relatively small sample size, which may limit the generalizability of the findings. In addition, the inferred evolutionary trends, including convergent evolution, should be interpreted with caution. These conclusions are based on a limited number of isolates from a single setting and may not fully represent broader population-level dynamics. Furthermore, mutations were observed only in *acrB*, with no mutations identified in *acrA* and *tolC* among the study isolates, although such mutations have been reported in a few global isolates. The expression of *acrA* and *tolC* and the functional assembly of the *acrAB–tolC* efflux system were not evaluated, and structural analysis was not performed. These limitations may affect the mechanistic interpretation of resistance and will be addressed in future studies.

In conclusion, this study provides mechanistic insights into azithromycin resistance in *S.* Typhi, suggesting that *acrB*_R717L/Q mutations in H58 lineages are associated with high-level resistance. The observed structural variability and conformational deviation may influence protein dynamics and potentially contribute to altered efflux-related function. However, direct functional assays to measure efflux activity were not performed, and therefore, the proposed impact of these mutations requires further experimental validation. Future studies should include (1) functional assays using isogenic mutants, (2) prospective multicentre studies linking genotype with clinical outcomes, development of rapid molecular assays for *acrB*_R717 detection, and (3) evaluation of cross-resistance to other macrolides. These findings support integrating chromosomal and plasmid-mediated resistance markers into genomic surveillance, with targeted *acrB*_R717 detection aiding early identification of high-risk isolates and informing clinical management. Overall, strengthening surveillance systems, promoting judicious antibiotic use and implementing preventive measures will be critical to controlling the spread of drug-resistant *S.* Typhi.

## Data Availability

The datasets presented in this study are available in online repositories. They can be retrieved under the repository name “Azithromycin resistance *Salmonella* Typhi in India” with the accession number PRJEB98498.

## Footnotes

1
https://github.com/ncbi/SKESA

2
https://github.com/typhoidgenomics/genotyphi

## Author’s note

This publication is part of the Ph.D. thesis of The Tamil Nadu Dr. M.G.R. Medical University.

## References

[ref1] Basnyat B. Qamar F. N. Rupali P. Ahmed T. Parry C. M. (2021). Enteric fever. BMJ Clin. Update. 372:n437. doi: 10.1136/bmj.n437 PMC7907991 33637488

[ref2] Carey M. E. Jain R. Yousuf M. Maes M. Dyson Z. A. Thu T. N. . (2021). Spontaneous emergence of azithromycin resistance in independent lineages of *Salmonella* Typhi in northern India. Clin. Infect. Dis. 72, e120–e127. doi: 10.1093/cid/ciaa1773, 33515460 PMC7935384

[ref3] Chavan S. Angadi K. (2025). A rise in azithromycin resistance among *Salmonella* isolates in India: a comprehensive review. Discov. Soc. Sci. Health 5:132. doi: 10.1007/s44155-025-00296-0

[ref4] Clinical and Laboratory Standards Institute. (2024). Performance Standards for Antimicrobial Susceptibility Testing. 34th ed. CLSI supplement M100. CLSI guidelines: CLSI.

[ref5] Croucher N. J. Page A. J. Connor T. R. Delaney A. J. Keane J. A. Bentley S. D. . (2015). Rapid phylogenetic analysis of large samples of recombinant bacterial whole genome sequences using Gubbins. Nucleic Acids Res. 43, e15–e15. doi: 10.1093/nar/gku1196, 25414349 PMC4330336

[ref6] Dola N. Z. Shamsuzzaman S. M. Islam S. Rahman A. Mishu N. J. Nabonee M. A. (2022). Distribution of ciprofloxacin-and azithromycin-resistant genes among *Salmonella* Typhi isolated from human blood. Int J Appl Basic Med Res 12, 254–259. doi: 10.4103/ijabmr.ijabmr_17_22, 36726659 PMC9886149

[ref7] Duy P. T. Dongol S. Giri A. Nguyen To N. T. Dan Thanh H. N. Nhu Quynh N. P. . (2020). The emergence of azithromycin-resistant *Salmonella* Typhi in Nepal. JAC Antimicrob. Resist. 2:dlaa109. doi: 10.1093/jacamr/dlaa109, 34223059 PMC8210228

[ref8] Farishta S. Faryal R. Waqas M. Ali M. Uppal R. Salman M. . (2025). Mechanisms of azithromycin resistance in *Salmonella typhi*: molecular insights from dynamics behavior to clinical implications. J. Biomol. Struct. Dyn. 44, 1158–1177. doi: 10.1080/07391102.2025.2524407, 40607646

[ref9] Feldgarden M. Brover V. Gonzalez-Escalona N. Frye J. G. Haendiges J. Haft D. H. . (2021). AMRFinderPlus and the reference gene catalog facilitate examination of the genomic links among antimicrobial resistance, stress response, and virulence. Sci. Rep. 11:12728. doi: 10.1038/s41598-021-91456-0 34135355 PMC8208984

[ref10] Hooda Y. Sajib M. S. Rahman H. Luby S. P. Bondy-Denomy J. Santosham M. . (2019). Molecular mechanism of azithromycin resistance among typhoidal *Salmonella* strains in Bangladesh identified through passive pediatric surveillance. PLoS Negl. Trop. Dis. 13:e0007868. doi: 10.1371/journal.pntd.0007868 31730615 PMC6881056

[ref11] Jiang W. Lacroix J. Luo Y. L. (2022). Importance of molecular dynamics equilibrium protocol on protein-lipid interaction near channel pore. Biophys. Rep. 2:100080. doi: 10.1016/j.bpr.2022.100080, 36425669 PMC9680783

[ref12] Kirchhelle C. Dyson Z. A. Dougan G. (2019). A biohistorical perspective of typhoid and antimicrobial resistance. Clin. Infect. Dis. 69, S388–S394. doi: 10.1093/cid/ciz556, 31612939 PMC6792120

[ref13] Kozlov A. M. Darriba D. Flouri T. Morel B. Stamatakis A. (2019). RAxML-NG: a fast, scalable and user-friendly tool for maximum likelihood phylogenetic inference. Bioinformatics 35, 4453–4455. doi: 10.1093/bioinformatics/btz305, 31070718 PMC6821337

[ref14] Lee J. Cheng X. Jo S. MacKerell A. D. Klauda J. B. Im W. (2016). CHARMM-GUI input generator for NAMD, GROMACS, AMBER, OpenMM, and CHARMM/OpenMM simulations using the CHARMM36 additive force field. Biophys. J. 110:641a. doi: 10.1016/j.bpj.2015.11.3431 PMC4712441 26631602

[ref15] Letunic I. Bork P. (2024). Interactive tree of life (iTOL) v6: recent updates to the phylogenetic tree display and annotation tool. Nucleic Acids Res. 52, W78–W82. doi: 10.1093/nar/gkae268, 38613393 PMC11223838

[ref16] Murzyn K. Róg T. Pasenkiewicz-Gierula M. (2005). Phosphatidylethanolamine-phosphatidylglycerol bilayer as a model of the inner bacterial membrane. Biophys. J. 88, 1091–1103. doi: 10.1529/biophysj.104.048835, 15556990 PMC1305115

[ref17] Nabarro L. E. McCann N. Herdman M. T. Dugan C. Ladhani S. Patel D. . (2022). British infection association guidelines for the diagnosis and management of enteric fever in England. J. Infect. 84, 469–489. doi: 10.1016/j.jinf.2022.01.014 35038438

[ref18] Piovani D. Figlioli G. Nikolopoulos G. K. Bonovas S. (2024). The global burden of enteric fever, 2017–2021: a systematic analysis from the global burden of disease study 2021. EClinicalMedicine 77:102883. doi: 10.1016/j.eclinm.2024.102883 39469533 PMC11513656

[ref19] Rodrigues C. H. Pires D. E. Ascher D. B. (2018). DynaMut: predicting the impact of mutations on protein conformation, flexibility and stability. Nucleic Acids Res. 46, W350–W355. doi: 10.1093/nar/gky300, 29718330 PMC6031064

[ref20] Souvorov A. Agarwala R. Lipman D. J. (2018). SKESA: strategic k-mer extension for scrupulous assemblies. Genome Biol. 19:153. doi: 10.1186/s13059-018-1540-z, 30286803 PMC6172800

[ref21] Tagg K. A. Kim J. Y. Henderson B. Birhane M. G. Snyder C. Boutwell C. . (2024). Azithromycin-resistant mph (a)-positive *Salmonella enterica* serovar Typhi in the United States. J. Glob. Antimicrob. Resist. 39, 69–72. doi: 10.1016/j.jgar.2024.08.005 39173740 PMC11663695

[ref22] Wong V. K. Baker S. Connor T. R. Pickard D. Page A. J. Dave J. . (2016). An extended genotyping framework for *Salmonella enterica* serovar Typhi, the cause of human typhoid. Nat. Commun. 7:12827. doi: 10.1038/ncomms12827 27703135 PMC5059462

[ref23] Yoshida C. E. Kruczkiewicz P. Laing C. R. Lingohr E. J. Gannon V. P. Nash J. H. . (2016). The *Salmonella* in silico typing resource (SISTR): an open web-accessible tool for rapidly typing and subtyping draft *Salmonella* genome assemblies. PLoS One 11:e0147101. doi: 10.1371/journal.pone.0147101 26800248 PMC4723315

